# Supplementary material for: SARS-CoV-2 Gut-Targeted Epitopes: Sequence Similarity and Cross-Reactivity Join Together for Molecular Mimicry
Source: Biomedicines. 2023 Jul 7;11(7):1937. doi: 10.3390/biomedicines11071937 (PMC10376948; doi:10.3390/biomedicines11071937)
Supplement: Supplementary file 1 [file biomedicines-11-01937-s001.zip › biomedicines-2477779-supplementary.pdf]

Table S1: Sequence similarity between additional SARS-CoV-2 proteins and enteric self-antigens related to ADs

Human Protein (Autoimmune-related Disease) [1]	SARS-CoV-2 Protein	Human VS SARS-CoV-2	Ln	Identity %	Similarity %	Score [2]
Cytochrome P450 2D6 (AIH, CD)	ORF1ab polyprotein	AFLPFSAG<br>AFLPFAMG	8	75	87.5	33
Myosin-11 (AIH)	ORF1ab polyprotein	ASSDKFVA<br>AGSDKGVA	8	75	75	24
	ORF1ab polyprotein	LEEEEAAR<br>LNLEEAAR	8	75	75	24
	ORF1ab polyprotein	PKGFMDGK<br>PKGFCDLK	8	75	75	30
	ORF1ab polyprotein	KQKDKKLK<br>KQDDKKIK	8	75	87.5	32
Dihydrolipoyllysine-residue acetyltransferase component of pyruvate dehydrogenase complex, mitochondrial (PBC)	ORF1ab polyprotein	LVPADNEK<br>LVPGFNEK	8	75	75	28
Small nuclear ribonucleoprotein Sm D1 (PBC)	ORF1ab polyprotein	LPLDTLLVD<br>LPTGTLLVD	9	77.8	77.8	32
Protein-glutamine gamma-glutamyltransferase 2 (CD)	ORF8 protein	QPQPFVVD<br>QHQPYVVD	8	75	87.5	32
	ORF1ab polyprotein	PFPASTGY<br>PFVVSTGY	8	75	75	33
	ORF1ab polyprotein	QPEEQPT<br>QPLEQPT	7	85.7	85.7	31
	ORF1ab polyprotein	EQKTVEIP<br>EQKIAEIP	8	75	75	30
	ORF1ab polyprotein	LLNLNLP<br>LSNLNLP	7	85.7	85.7	29
	ORF10 protein	RNYEASVD<br>RNYIAQVD	8	75	75	29
Matrin-3 (AIH)	ORF1ab polyprotein	FDSEYERMG<br>FDSEYCRHG	9	77.8	77.8	33
	ORF1ab polyprotein	QKLKKFLN<br>KKLKKSLN	8	75	87.5	28
	ORF1ab polyprotein	KVDKIEEL<br>KAYKIEEL	8	75	75	25
	ORF1ab polyprotein	IATGGAVF<br>IAFGGCVF	8	75	75	28
Formimidoyltransferase-cyclodeaminase (AIH)	ORF8 protein	VGARSAAP<br>VGARKSAP	8	75	87.5	31
Lipoamide acyltransferase component of branched-chain alpha-keto acid	ORF1ab polyprotein	PPKPKDMT<br>PGIPKDMT	8	75	75	30
	Spike glycoprotein	IDLTELVK	8	75	75	24

dehydrogenase complex, mitochondrial (AIH)		IDLQELGK				
E3 ubiquitin-protein ligase TRIM21 (AIH)	ORF1ab polyprotein	KLEVEIAI<br>KLNEEIAI	8	75	75	24
	ORF1ab polyprotein	LDPDTANP<br>LKVDTANP	8	75	75	29
	Spike glycoprotein	PRRQASSA<br>PRRAASVA	8	75	75	26
	nucleocapsid phosphoprotein	QSGERSG<br>QNGERSG	7	85.7	100	32
	ORF1ab polyprotein	GRSGETSG<br>GRSGETLG	8	87.5	87.5	35
Protein disulfide-isomerase A3 (AIH)	ORF3a protein	LFPGVALL<br>LIVGVALL	8	75	75	24
	Nucleocapsid phosphoprotein	EEKPKKKKKA<br>EPKKDKKKKA	10	70	70	31
Dihydrolipoyllysine-residue succinyltransferase component of 2-oxoglutarate dehydrogenase complex, mitochondrial (AIH)	Spike glycoprotein	IDDTTKEV<br>IDDTTDAV	8	75	75	28
Smoothelin (AIH)	ORF1ab polyprotein	AIRRVRA<br>AIRHVRA	7	85.7	85.7	26
	ORF1ab polyprotein	EKLEKEGA<br>ENLTKEGA	8	75	75	28
O-phosphoseryl-tRNA(Sec) selenium transferase (AIH,PBC)	ORF1ab polyprotein	TDLKAVEA<br>TTLKGVEA	8	75	75	26
	ORF1ab polyprotein	GDELRTD<br>GDELGTD	7	85.7	85.7	30
Nuclear pore membrane glycoprotein 210 (PBC)	Spike glycoprotein	FHAHSSVLN<br>FGAISSVLN	9	77.8	77.8	27
	ORF3a protein	AASPIITL<br>SASKIITL	8	75	87.5	25
	nucleocapsid phosphoprotein	KGFLASGS<br>KGFYAEGS	8	75	75	30
	ORF1ab polyprotein	LATVLTSL<br>LLTILTSL	8	75	87.5	28
	ORF1ab polyprotein	LVAVPLG<br>LVAVPTG	7	85.7	85.7	28
	ORF1ab polyprotein	DESGQKKL<br>DESGEFKL	8	75	87.5	29
Cytoskeleton-associated protein 5 (IBD)	ORF1ab polyprotein	LVKALKKV<br>LAKALRKV	8	75	87.5	28
	ORF1ab polyprotein	VLGDSKNN<br>VTGDSCNN	8	75	75	28
	ORF1ab	KAVNPFLA	8	75	75	27

	polyprotein	KLVNKFLA				
Noggin (UC)	ORF1ab polyprotein	DLNETLL<br>DLNETLV	7	85.7	100	31
Sucrase-isomaltase, intestinal (Crohn's disease)	Spike glycoprotein	PNKRSFI<br>PSKRSFI	7	85.7	100	32
	ORF1ab polyprotein	LAFQTVK<br>VAFQTVK	7	85.7	100	30
	ORF1ab polyprotein	TSIGPLV<br>TSFGPLV	7	85.7	85.7	30
	ORF1ab polyprotein	YVIILDPA<br>YVYIGDPA	8	75	75	27

[1]Human proteins that are implicated in enteric autoimmune diseases: autoimmune hepatitis (AIH), celiac disease (CD), primary biliary cholangitis (PBC), ulcerative colitis (UC), and inflammatory bowel disease (IBD).

[2]**Score** is based on BLOSUM62 substitution matrix between amino acids.

Table S2: The functionality of additional enteric antigens and their corresponding similar sequences

Shared Peptides	Enteric protein and potential function/pathogenesis	AD[1]	Ref
AFLPFSAG	**Cytochrome P450 2D6 (CYP2D6, UniProt:P10635)**<br>Has been recognized as the major autoantigen in type 2 AIH. In patients with AIH-2, the target for anti-LKM-1 antibodies has been identified as the 2D6 isoform of the large cytochrome P450 enzyme family.		[62–65]
ASSDKFVA<br>LEEEEAAR<br>PKGFMDGK<br>KQKDKKLK	**Myosin-11 (SMMHC, UniProt:P35749)**<br>Autoantibodies to non-muscle myosin heavy chain were reported in patients with chronic liver diseases. Patients presenting with ANA and/or smooth muscle antibodies (SMA), account for about 80% of cases of AIH.	**AIH**	[68,69]
LVPADNEK	**Dihydrolipoyllysine-residue acetyltransferase component of pyruvate dehydrogenase complex, mitochondrial (PDC-E2, UniProt:P10515)**<br>PBC patients have been characterized to have autoreactive T-cell and B-cell responses directed at self-PDC-E2. The diagnosis of PBC is readily reached by the detection of specific AMA directed against PDH-E2.	**PBC**	[73,74]
LPLDTLLVD	**Small nuclear ribonucleoprotein SmD1 (SmD1, UniProt:P62314)**<br>This protein autoantigen falls into the classical autoantibody categories of autoimmune liver diseases: ANA (anti-nuclear autoantibodies), SMA (anti-smooth muscle autoantibodies), AMA, and LKM (liver-kidney microsomal autoantigens)..	**AIH**<br>**PBC**	[148]
QPQPFVVD<br>PFPASTGY<br>QPEEQPT<br>EQKTVEIP<br>LLNLNLP<br>RNYEASVD	**Protein-glutamine gamma-glutamyltransferase 2 (TG2, UniProt:P21980)**<br>TG2 is a ubiquitous enzyme that physiologically exerts multiple functions, mostly linked to matrix assembly and tissue repair. It is considered as the autoantigen in CD and the anti-TG2 autoantibodies are the prime serological marker of CD.	**CD**	[25,149]
FDSEYERMG<br>QKLKKFLN<br>KVDKIEEL<br>IATGGAVF	**Matrin-3 (UniProt: P43243)**<br>A major protein that makes up the fibrogranular network of the nuclear matrix. Used as a diagnostic and prognostic marker in hepatic tumor progression.	**AIH**	[150]
VGARSAAP	**Formimidoyltransferase-cyclodeaminase (FTCD, UniProt:O95954)**<br>This enzyme is a liver-specific antigen recognized by the sera of patients with autoimmune hepatitis.	**AIH**	[151]
PPKPKDMT<br>IDLTELVK	**Lipoamide acyltransferase component of branched-chain alpha-keto acid dehydrogenase complex, mitochondrial (BCOADC-E2, UniProt:P11182)**<br>A 52 kDa mitochondrial autoantigen has been identified as the E2 subunit of the 2-oxo-acid dehydrogenase complex in PBC diagnosis.	**PBC**	[74,152]
KLEVEIAI<br>LDPDTANP<br>PRRQASSA<br>QSGERSG<br>GRSGETSG	**E3 ubiquitin-protein ligase TRIM21 (52 kDa Ro, UniProt:P19474)**<br>Patients with AIH frequently express autoantibodies against soluble liver antigen/liver pancreas (anti-SLA/LP) and/or against 52 kDa Ro protein (anti-Ro52).	**AIH**	[153–155]
LFPGVALL<br>EEKPKKKKKA	**Protein disulfide-isomerase A3 (ERp57, UniProt:P30101)**<br>Antibodies against epitopes of ERp57were detected in Liver kidney microsomal antibody type 1 positive sera in AIH patients.	**AIH**	[156]
IDDTTKEV	**Dihydrolipoyllysine-residue succinyltransferase component of 2-oxoglutarate dehydrogenase complex, mitochondrial (OGDC-E2, UniProt: P36957)**<br>Associated with several ADs	**AIH**<br>**PBC**	[152,157,158]
AIRRVRA<br>EKLEKEGA	**Smoothelin (UniProt:P53814)**<br>A structural protein that is found exclusively in contractile smooth muscle cells. Part of the anti-smooth muscle antibodies in AIH.	**AIH**	[150,159]

TDLKAVEA<br>GDELRTD	**O-phosphoseryl-tRNA(Sec) selenium transferase (SLA, UniProt:Q9HD40)**<br>Anti-SLA autoantibodies test is a diagnostic marker for HIA in patients who are negative for other autoantibodies and may otherwise be misdiagnosed. In AIH, the presence of anti-SLA autoantibodies is associated with reduced overall survival.	**AIH**<br>**PBC**	[160,161]
FHAHSSVLN<br>AASPIITL<br>KGFLASGS<br>LATVLTSL<br>LVAVPLG<br>DESGQKKL	**Nuclear pore membrane glycoprotein 210 (gp210, UniProt:Q8TEM1)**<br>Anti-nuclear envelope-gp210 antibodies are specifically detected in 20-30% of PBC patients; the presence of anti-gp210 antibodies predicts a more severe disease course.	**PBC**	[162,163]
LVKALKKV<br>VLGDSKNN<br>KAVNPFLA	**Cytoskeleton-associated protein 5 (Ch-TOG, UniProt:Q14008)**<br>Involved in cell division, and tight junctional functional integrity. Suggested to have a role in IBD pathogenesis and colorectal cancer spreading in Crohn's disease.	**IBD**	[137,164]
DLNETLL	**Noggin (NOG, UniProt:Q13253)**<br>Essential for cartilage morphogenesis and joint formation. Involved in the arthropathy in Crohn's disease.	**IBD**	[165]
PNKRSFI<br>LAFQTVK<br>TSIGPLV<br>YVIILDPA	**Sucrase-isomaltase, intestinal (SI, UniProt:P14410)**<br>A marked specific decrease in sucrase-isomaltase gene expression in villous enterocytes in acutely inflamed Crohn's ileum as compared to adjacent uninflamed ileum and normal ileum was reported.	**IBD**	[166]

[1] Autoimmune diseases: autoimmune hepatitis (AIH), celiac disease (CD), primary biliary cholangitis (PBC), and inflammatory bowel disease (IBD - including ulcerative colitis and Crohn’s disease).

Table S3: Additional similar epitopes' sequence with immunoreactive validation in experimental assays in IEDB

Human Epitope	SARS-CoV-2 Epitope	Human vs SARS-CoV-2	IEDB Human Assays' References	IEDB SARS-CoV-2 Assays' References
GHFVKPE**AFLPFSAG**RRACL	LYEN**AFLPFAMG**	AFLPFSAG<br>AFLPFAMG	Tcell [76]	Bcell(IgM) [82]
N**ASSDKFVA**DL	HFG**AGSDKGVA**PGTA	ASSDKFVA<br>AGSDKGVA	HLA-I [93]; HLA-II [90]	Bcell(IgM) [82]
DLEEQ**LEEEEAAR**QKLQLEKVT	THG**LNLEEAAR**YMRS	LEEEEAAR<br>LNLEEAAR	HLA-II [93]	Bcell(IgM) [82]
NAI**PKGFMDGK**QA	IDHPN**PKGFCDLK**GK	PKGFMDGK<br>PKGFCDLK	HLA-II [93]	Bcell(IgA) [78]
SL**KQKDKKLK**E	R**KQDDKKIK**ACV	KQKDKKLK<br>KQDDKKIK	HLA-II [93]	Bcell(IgM) [82]
ILAIGASEDK**LVPADNEK**GFDVA	**LVPGFNEK**THVQLSL	LVPADNEK<br>LVPGFNEK	HLA-II [93]	Bcell(IgA) [78]
FILPDS**LPLDTLLVD**VEPK	RQW**LPTGTLLVD**SDL	LPLDTLLVD<br>LPTGTLLVD	HLA-I; HLA-II [167]	Bcell(IgA) [78]
E**QPQPFVVD**WIQ	T**QHQPYVVD**DPCPIH	QPQPFVVD<br>QHQPYVVD	Bcell(IgA) [168]	DRB1*15:01;Tcell [77]; Bcell(IgG) [169]
QPE**PFPASTGY**Q	DGV**PFVVSTGY**HFRE	PFPASTGY<br>PFVVSTGY	Bcell(IgA) [168]	Bcell(IgA) [78]
**QPEEQPT**VSYNG	DL**QPLEQPT**SEA	QPEEQPT<br>QPLEQPT	Bcell(IgA) [168]	Bcell(IgM) [82]
T**EEQKTVEIP**	V**EQKIAEIP**KEE	EQKTVEIP<br>EQKIAEIP	HLA-I [97]; DQA1*02:01/ DQB1*02:02 [170]	Bcell(IgM) [82]
PEQ**LLNLNLP**EQ	FDTRV**LSNLNLP**GCD	LLNLNLP<br>LSNLNLP	Bcell(IgA) [168]	Bcell(IgM) [82]
LTLHFEG**RNYEASVD**	MNS**RNYIAQVD**V	RNYEASVD<br>RNYIAQVD	Bcell(IgA) [171]	Bcell(IgM) [82]
HK**FDSEYERMG**	T**FDSEYCRHG**TCER	FDSEYERMG<br>FDSEYCRHG	HLA-I [83]; DRB1*04:05 [93]	Bcell(IgM) [82]
SLPHY**QKLKKFLN**KLAEERRQ	EVVL**KKLKKSLN**VAK	QKLKKFLN<br>KKLKKSLN	HLA-II [93]	Bcell(IgM) [82]
KLK**KVDKIEEL**	SD**KAYKIEEL**FYSYA	KVDKIEEL	A*02:02 [79];	Bcell(IgA) [78]

		KAYKIEEL	HLA-II [172]	
A**IATGGAVF**	**IAFGGCVF**SYVGCHN	IATGGAVF	C*12:02 [173]; DQA1*05:05/ DQB1*03:01 [170]; A*01:01 [174]; A*02:01 [175]; B*15:02 [96]; DQ [99]	Bcell(IgA) [78]
		IAFGGCVF		
RAFVGE**VGARSAAP**GGGSV	YIR**VGARKSAP**LIEL	VGARSAAP	HLA-II [176]	Tcell [94]; Bcell(IgG) [169]
		VGARKSAP		
VEIMPP**PPKPKDMT**VPILV	CVDI**PGIPKDMT**YRR	PPKPKDMT	HLA-II [90]	Bcell(IgA) [78]
		PGIPKDMT		
**IDLTELVK**LREELKPIAF	NLNESL**IDLQELGK**YE	IDLTELVK	HLA-II [93]	A*11:01 [91]
		IDLQELGK		
ELAE**KLEVEIAI**KRADWK	**KLNEEIAI**I	KLEVEIAI	Bcell(IgG) [153]	A*02:01 [177]; Tcell [96]; Bcell(IgA) [78]
		KLNEEIAI		
T**LDPDTANP**WLILSE	**LKVDTANP**KTPKYKF	LDPDTANP	Bcell(IgG) [178]	Bcell(IgA) [78]; Tcell [94]
		LKVDTANP		
GI**PRRQASSA**VR	S**PRRAASVA**SQ	PRRQASSA	Bcell(IgG) [179]; HLA-II [93]	HLA-II [95]
		PRRAASVA		
G**QSGERSG**RSGS	SN**QNGERSG**ARSKQR	QSGERSG	Bcell(IgG) [179]	Tcell [88]; Bcell(IgA) [78]
		QNGERSG		
AR**GRSGETSG**HS	GIQY**GRSGETLG**VLV	GRSGETSG	Bcell(IgG) [179]; HLA-I [79]	Bcell(IgA) [78]
		GRSGETLG		
A**LFPGVALL**	ASLPFGW**LIVGVALL**	LFPGVALL	A*02:01 [180]; B*27:05 [181]; HLA-I [182]	Bcell(IgA) [78]; HLA [183]
		LIVGVALL		
NPPVIQ**EEKPKKKKKA**QE	**TEPKKDKKKKA**DETQ	EEKPKKKKKA	DR [184]; DRB5*01:01 [185]; HLA-II [93]	Tcell [88]; Bcell(IgG) [85]
		EPKKDKKKKA		
V**IDDTTKEV**	RD**IDDTTDAV**	IDDTTKEV	HLA-I [186]	A*02:01 [187]
		IDDTTDAV		
ERKLIRAAIRRVRAQE	NMFITREE**AIRHVRA**	AIRRVRA	HLA-II [167]	Tcell [94]; Bcell(IgA) [78]
		AIRHVRA		
**EKLEKEGA**AGSPGGPR	EFCGT**ENLTKEGA**TT	EKLEKEGA	DRB1*04:01	Bcell(IgA) [78]

		ENLTKEGA	[170];<br>HLA-II [93]	
ELR**TDLKAVEA**KVQELGPDC	**TTLKGVEA**VMYMGTL	TDLKAVEA<br>TTLKGVEA	Tcell [161]	Bcell(IgA) [78]
IENVLE**GDELRTD**LKAVEAK	**LGDELGTD**PYEDFQ	GDELRTD<br>GDELGTD	Tcell [161]	Bcell(IgM) [82]
V**FHAHSSVLN**F	QLSSN**FGAISSVLN**D	FHAHSSVLN<br>FGAISSVLN	HLA-I [188];<br>DQ [99]	HLA [95];<br>Tcell [189];<br>Bcell(IgA) [78]
**AASPIITL**V	**SASKIITL**K	AASPIITL<br>SASKIITL	HLA-I [188];<br>C*12:02 [173]	A*11:01 [190];<br>Tcell [191];<br>Bcell(IgM) [192]
HVDE**KGFLASGS**MIGT	QGTTLP**KGFYAEGS**R	KGFLASGS<br>KGFYAEGS	HLA-II [176]	Bcell(IgM) [89]
**LATVLTSL**	**LLLTILTSL**	LATVLTSL<br>LLTILTSL	B*51:01 [193]	A*02:01 [177]
A**LVAVPLG**MTV	FSTGVN**LVAVPTG**YV	LVAVPLG<br>LVAVPTG	A*02:01<br>[194]	Bcell(IgA) [78]
D**DESGQKKL**HGLQAILV	YLF**DESGEFKL**	DESGQKKL<br>DESGEFKL	DR [99];<br>B*07:02<br>[175];<br>HLA-II<br>[172];<br>DR1 [195];<br>A*01:01<br>[174]	HLA-I [83];<br>Tcell [196];<br>Bcell(IgM) [82]
DYAD**LVKALKKV**VGKDTN	**MLAKALRKV**	LVKALKKV<br>LAKALRKV	HLA-II [197]	A*02:01; HLA<br>[177];<br>Bcell(IgA) [78]
**TVLGDSKNNV**	**EVTGDSCNN**YMLTYN	VLGDSKNN<br>VTGDSCNN	HLA-I [198]	Tcell [77];<br>Bcell(IgA) [78]
VGE**KAVNPFLA**DVDK	**KLVNKFLA**L	KAVNPFLA<br>KLVNKFLA	DPA1*02:01/<br>DPB1*01:01<br>[199]	Tcell [200]
FDPKEK**DLNETLL**RSLLGGHYD	NTLN**DLNETLV**T	DLNETLL<br>DLNETLV	Bcell(IgG)<br>[165]	Bcell(IgM) [82]
**FPNKRSFI**LTRSTFAGSG	LPDPSK**PSKRSFI**EDLLFNKVT	PNKRSFI<br>PSKRSFI	DRB1*07:01<br>[93]	HLA [95];<br>Tcell [94];<br>Bcell(IgG) [85]
TT**LAFQTVK**	AALTNN**VAFQTVK**PGN	LAFQTVK<br>VAFQTVK	A*11:01 [93]	Bcell(IgG) [201]

VIRKSNGKTLFD**TSIGPLV**YSDQY	**TSFGPLV**RK	TSIGPLV<br>TSFGPLV	HLA-II [93]	A*03:01 [190];<br>Tcell [191];<br>Bcell(IgA) [78]
K**YVIILDPA**ISIG	AKH**YVYIGDPA**QLPA	YVIILDPA<br>YVYIGDPA	DRB1*13:03;<br>HLA-II [93]	Bcell(IgM) [202]
